# Simultaneous-Fault Diagnosis of Gearboxes Using Probabilistic Committee Machine

**DOI:** 10.3390/s16020185

**Published:** 2016-02-02

**Authors:** Jian-Hua Zhong, Pak Kin Wong, Zhi-Xin Yang

**Affiliations:** Department of Electromechanical Engineering, University of Macau, Macao, China; zjheme@gmail.com (J.-H.Z.); fstpkw@umac.mo (P.K.W.)

**Keywords:** simultaneous-fault diagnosis, Hilbert-Huang transform, pairwise-coupling probabilistic committee machine

## Abstract

This study combines signal de-noising, feature extraction, two pairwise-coupled relevance vector machines (PCRVMs) and particle swarm optimization (PSO) for parameter optimization to form an intelligent diagnostic framework for gearbox fault detection. Firstly, the noises of sensor signals are de-noised by using the wavelet threshold method to lower the noise level. Then, the Hilbert-Huang transform (HHT) and energy pattern calculation are applied to extract the fault features from de-noised signals. After that, an eleven-dimension vector, which consists of the energies of nine intrinsic mode functions (IMFs), maximum value of HHT marginal spectrum and its corresponding frequency component, is obtained to represent the features of each gearbox fault. The two PCRVMs serve as two different fault detection committee members, and they are trained by using vibration and sound signals, respectively. The individual diagnostic result from each committee member is then combined by applying a new probabilistic ensemble method, which can improve the overall diagnostic accuracy and increase the number of detectable faults as compared to individual classifiers acting alone. The effectiveness of the proposed framework is experimentally verified by using test cases. The experimental results show the proposed framework is superior to existing single classifiers in terms of diagnostic accuracies for both single- and simultaneous-faults in the gearbox.

## 1. Introduction

In the rotating machinery, gearboxes are widely used to transmit power from the prime mover to the load. If any failure occurs in the gearbox, it may interrupt normal machine operation and endanger users. Consequently, it is of great significance to develop a reliable and accurate intelligent system to diagnose the main components of the gearbox, such as gears and bearings. There are two main challenges in gearbox diagnosis. One is the existence of simultaneous faults, that is, multiple single faults that appear concurrently. The other is that no unique sensor can detect all the machine faults. To accurately detect more faults, many kinds of sensors and signals may be involved at the same time. However, it is difficult to analyze different kinds of signals simultaneously and make a decision. In the [1,2,3,4,5,6,7], various gearbox diagnostic systems have been proposed. In these systems, the fault diagnosis procedures are mainly divided into two stages: (1) signal processing and (2) fault identification/classification.

The existing problems in signal processing of these systems are that the signals usually contain high-dimensional data and suffer from background noise interference, which degenerates the accuracy and fault identification time. Besides, the gearbox usually has many rotating components working together, such as bearings, gears and spindles, so the diagnosis of the gearbox is a simultaneous fault problem. In traditional gearbox fault diagnostic methods, simultaneous faults are usually considered as an independent label for the classifier, which will result in a high cost in acquiring exponentially increased simultaneous fault signals. For example, with *d* single-faults (labels) and one normal condition, there are 2*^d^* − (*d* + 1) artificial simultaneous fault labels [8,9,10]. To solve this problem, an effective signal de-noising method and a proper feature extraction technique which can find the single fault pattern features in simultaneous fault patterns are studied together.

Currently, some methods, including spectral subtraction, least squares, and wavelet threshold methods, are widely used for signal de-nosing [11,12]. In order to effectively de-noise the non-stationary signals of a gearbox, a soft threshold method based on the discrete wavelet transform (DWT) is adopted in this study due to its popularity.

References [8,9,10] reported that a simultaneous fault symptom can be identified by analyzing the single fault patterns only if the classifier is trained by using a proper feature extraction technique, so that it can save a lot of resources to collect a large combination of simultaneous fault training data. Existing techniques to select a proper feature extraction technique are reviewed here. At present, there exist many methods to extract features from fault signals, such as Fourier transform, short time Fourier transform, and wavelet transform. The Fourier transform is only suitable for analyzing stationary signals. However, the signals of rotating gears and bearings are non-stationary, which makes the Fourier transform unsuitable for this application. The time-frequency analysis methods, such as short time Fourier transform (STFT) and wavelet transform, can process non-stationary signals, but they all have limitations. STFT has a limitation in non-stationary signal processing because of its use of a fixed time window which makes it impossible to achieve good resolution in the time and frequency domains at the same time. The drawback of the wavelet transform is that it suffers from the effect of the energy leakage because any signal which does not well correlate with the shape of wavelet basis function will be masked or completely ignored. In contrast to STFT and the wavelet transform, the Hilbert-Huang transform (H-HT) is the latest time-frequency signal processing technique to analyze nonlinear and non-stationary signals. The first step of a typical H-HT process is to employ the empirical mode decomposition (EMD) algorithm to decompose a complicated signal into a series of intrinsic mode functions (IMFs), which contains the local characteristics of the original signal at different time scales, and then a Hilbert transform is applied to each intrinsic mode function (IMF) for Hilbert spectrum analysis. The high time-frequency resolution of the H-HT method can effectively describe the rules of the changing frequency compositions with time, which is a good approach for analyzing non-stationary signals. Even though H-HT has been applied to many applications, particularly in fault detection and diagnosis [13,14], it has some disadvantages: (1) the issue of mode mixing; and (2) the redundant intrinsic mode functions easily appear at low frequency, which can cause the distortion of the processed result [15]. To overcome these disadvantages, this study applies ensemble empirical mode decomposition (EEMD), an improved EMD method, to deal with the mode mixing problem, and uses the correlation coefficient method to eliminate the redundant IMFs. The EEMD-based H-HT is hereafter refered to as HHT. It is well-known that different fault conditions show different amplitude- and phase-frequency characteristics in the frequency domain. In other words, fault signal energies in some frequency bands may be enhanced, while the others are restrained. It is reasonable to assume that there are certain corresponding relationships between the signal energy changes in the frequency bands and the fault phenomena. Therefore, on the basis of HHT, energy patterns of the selected intrinsic mode function components are considered in this study to further extract representative fault features from the gearbox vibration and sound signals.

In [1,3,5], most of the existing fault classification systems for the rotating machinery are constructed by a single classifier which is trained based on one type of signal. However, a single classifier-based fault diagnostic system may not give reliable fault diagnostic results due to the fact that a universal classifier is difficult to develop, especially when the data available for training the classifier are not abundant. Furthermore, a single classifier can only be trained by one type of signal. Obviously, only one type of signal may not be able to cover all the faults. To let a fault classification system generate more reliable diagnostic result and diagnose more faults, this paper proposes a new probabilistic committee machine (PCM) to combine the diagnostic results from vibration and sound signals. From the gearbox point of view, vibration and sound signals are usually used to identify the faults because those signals are easily acquired and highly related to the conditions of the gearbox [16,17,18,19,20,21]. The committee machine concept involves combining results acquired by individual classifiers so as to obtain a group decision that is superior to any individual classifier acting alone [22,23,24], because a group decision is usually better than a single person’s decision.

Moreover, a proper classifier must be able to offer the probabilities of all possible faults so that the user can at least trace the other possible faults according to the rank of their probabilities when the fault(s) predicted by the classifier are incorrect. Therefore, it is logical to employ a probabilistic classifier for each member in the committee machine for simultaneous-fault diagnosis of the gearbox. Currently, there are two common probabilistic classifiers, the probabilistic neural network (PNN) [25,26] and relevance vector machine (RVM) [27,28] available in the relevant literature. The main drawback of PNN lies in the limited number of inputs because the complexity of the network and the training time are heavily related to the number of inputs. Hence, RVM is selected as a probabilistic classifier to build each committee member in this study. Generally, the aforementioned probabilistic classifiers are suitable to solve the binary classification. Nevertheless, most of the practical applications are multi-class classification problems. One-*versus*-all strategy is usually employed to fix the multi-class classification problem. However, this strategy does not consider the correlation between every pair of faults or labels, which was verified to produce a large region of indecision [29]. To solve the multi-class classification problem effectively and generate a probability, a suitable pairwise coupling strategy is adopted for the above probabilistic classifiers to generate a pairwise-coupled probabilistic neural network (PCPNN) and pairwise-coupled relevance vector machine (PCRVM).

After determining the methods of signal de-noising, feature extraction and committee members, there are still two major factors, the decision threshold *ε* and member weight *w*, affecting the system accuracy in the proposed framework. The probabilistic committee machine only produces the probability of occurrence of each fault. To determine the occurrence of the faults, a decision threshold must be applied to those probabilities (e.g., output probabilistic vector **P** = [0.35, 0.58, 0.48, 0.83], if *ε* = 0.5, fault labels (2, 4) are considered as faults). Besides, different committee members usually have various reliabilities, so a fair committee machine should assign different weights to their committee members. Hence, an efficient searching algorithm, particle swarm optimization (PSO) [30,31], to determine optimal member weights and decision threshold is considered in the proposed framework. Finally, a fair measure, *F*-measure, is employed to evaluate the performance of the proposed diagnostic framework.

In a nutshell, this paper proposes a new framework which can diagnose simultaneous faults in the gearbox while the framework is trained using only single-fault patterns. Besides, the proposed framework can provide probabilities of all possible faults to users to trace the other possible faults according to the rank of probabilities when the diagnostic result is incorrect. Furthermore, the proposed framework can generate a more reliable diagnostic result and diagnose more faults by simultaneously analyzing vibration and sound signals. Even though the authors also proposed a similar framework for simultaneous-fault diagnosis of automotive engines in [21], the proposed framework is targeted at the gearbox system. Moreover, the signal patterns used in this application are totally different from the ones in [21]. The proposed framework is designed based on vibration and sound signals rather than air ratio, ignition and acoustic signals in the previous framework. Besides, the engine signals acquired in [21] do not consider the issue of background noise which can degenerate the accuracy of the diagnostic system. Furthermore, the feature extraction and selection methods rely on EMD + domain knowledge and sample entropy, which are old, time-consuming, out of support from reference materials, and have a risk of mode-mixing. Finally, the objective function in [21] is not well-defined that cannot achieve good diagnostic accuracy. Therefore, the framework in [21] cannot be directly applied and is modified significantly to suit for the gearbox, particularly in the phases of data processing and feature selection. Table 1 summarizes the differences between the diagnostic framework in [21] and this study.

**Table 1:** Differences of diagnostic framework between reference [21] and this study.

Differences	Reference [21]	Present Study
Application	Automotive engine	Gearbox
Signal patterns	Air ratio, ignition and acoustic signals	Vibration and sound signals
Signal de-noising	None	Wavelet threshold
Feature extraction	EMD and domain knowledge	EEMD-based Hilbert-Huang transform and energy pattern
Feature selection (IMF selection)	Value of sample entropy	Correlation coefficient
Objective function	*F_me_* ∈ 0.925 ± 0.025	*F_me_* ≥ 0.9

This paper is organized as follows: Section 2 presents the proposed framework and related techniques. The experimental setup and data per-processing are discussed in Section 3. Section 4 discusses the experimental results and a comparison with other approaches. Finally, conclusions are given in Section 5.

## 2. Proposed Framework

**Figure 1:**
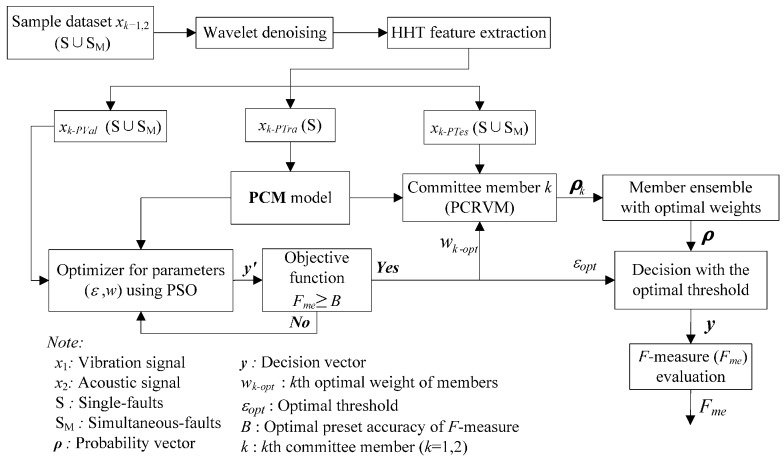
Proposed framework of gearbox simultaneous-fault diagnosis using probabilistic committee machine.

The proposed PCM framework for the gearbox simultaneous-fault diagnosis, evaluation approach and its construction method are illustrated in Figure 1. The framework consists of four sub-modules: (1) data processing; (2) probabilistic committee machine; (3) parameter optimization; and (4) performance evaluation. The details of the four sub-modules in the framework are discussed in the following sub-sections.

In this case study, signal features are extracted from two kinds of signals *x_k_* (*k* = 1, 2), including the vibration and sound signals, which are denoted as *x*_1_ and *x*_2_, respectively. Taking the vibration signal as an example, the signal *x*_1_, including both single-fault patterns (S) and simultaneous-fault patterns (S_M_), goes through de-noising and feature extraction. After the data processing, the processed dataset is divided into three independent groups, including validation dataset, training dataset, and test dataset which are named as *x*_1-*PTra*_, *x*_1-*PVal*_, and *x*_1-*PTes*_, respectively. The *x*_1-*PVal*_ and *x*_1*-PTes*_ involve the combination of both single-fault patterns and simultaneous-fault patterns, while *x*_1-*PTra*_ contains the single-fault patterns only. The divided datasets are used to train, validate, and test the proposed framework.

### 2.1. Data Processing

#### 2.1.1. Signal De-Noising

The acquired signals are display interference from the background noise. To decrease the interference, the acquired signals have to be de-noised. A discrete wavelet transform (DWT) technique, which is an effective de-noising technique for non-stationary signals [11,13], is selected in this paper. The DWT can be defined as:

(1) $$\mathrm{DWT}(s,R)=\frac{1}{\sqrt{2^{s}}}\int_{-\infty }^{\infty }x(t)\psi^{*}(\frac{t-2^{s}R}{2^{s}})dt$$

where *s* and *R* are integers, 2*^s^* and 2*^s^R* represent the scale and translation parameters respectively, *Ψ* represents the mother wavelet and Ψ^*^ is the complex conjugate of *Ψ*. The original signal in time-domain *x_k_* = *x*(*t*) goes through a set of low pass and high pass filters emerging as low frequency (approximations, a^*^) and high frequency (details, $d_{i}^{*}$) signals. Therefore, the original signal *x*(*t*) can be written as:

(2) $$x(t)=a_{n}^{*}+\sum_{i=1}^{n}d_{i}^{*}$$

The DWT-based de-noising technique is performed in three steps: (1) signal decomposition; (2) determination of the threshold and nonlinear shrinking coefficients; and (3) signal reconstruction. In the family of mother wavelets, the Daubechies wavelet (Db) is the most popular one and hence it is employed in this study. Moreover, the soft threshold signal is defined as sign(x(t))(|x(t)−T|), if |x(t)|>T, and otherwise is 0, where *T* denotes a universal threshold that equals to $\sqrt{2\log(length\;x(t))}$. The detail of the de-noising is described in Section 3.2.

#### 2.1.2. Feature Extraction Based on Hilbert-Huang Transform

The Hilbert-Huang transform (HHT) mentioned in this paper combines EEMD and the Hilbert transform. EEMD defines the true IMFs as the ensemble mean of trails, which consist of the decomposition of the signal plus a white noise of finite amplitude. In most cases, the range of the standard deviation is from 0.1 to 0.4 [32]. The EEMD algorithm [33] is given as follows:

(1) Initialize the number of ensemble *J*, the amplitude of the added white noise, and set *j* = 1.
(2) Perform the *j*th trial on the white noise-added signal. A white noise series with the given amplitude is added to the investigated signal:
  (3) $$x{´}_{j}=x\left(t\right)´+n_{j}$$
  where *n_j_* represents the *j*th added white noise series, *x(t)*’ is the de-noised signal and *x’_j_* denotes the noise-added signal of the *j*th trial.
(3) With the EMD method, the noise-added signal *x_j_* is decomposed into *I* IMFs as *c_i,j_*(*t*), for *i* = 1, 2, …, *I*, where *c_i,j_* represents the *i*th IMF of the *j*th trial, and *I* is the number of IMFs.
(4) If *j* < *J* then let *j* = *j* + 1. Repeat Steps 2 and 3 again and again, but with different white noise series each time until *j* = *J*.
(5) Calculate the ensemble mean $\bar{c_{i}}$ of *J* trials for each IMF:
  (4) $$\bar{c_{i}}=\frac{1}{J}\sum_{j=1}^{J}c_{i,j},\;\;i=1,2,...,I,\;j=1,2,...,J$$
(6) Report the mean $\bar{c_{i}}$ of the *I* IMFs as the final IMFs.

Applying the Hilbert transform to each IMF, and calculating the instantaneous frequency $\omega_{j}$(*t*) and amplitude *A_j_*(*t*), the Hilbert spectrum of *x*(*t*)’, H(ω,t), is then calculated by the following equation:

(5) H(ω,t)=Re∑j=1IAj(t)exp(i∫ωj(t)dt)

Accordingly, the marginal spectrum of Hilbert-Huang transform, *h*(ω), can be defined by an integrated spectrum with respect to time, *t*, *i.e.*:

(6) $$h(\omega )=\int_{0}^{l}H(\omega ,t)dt$$

where *h*(ω) reflects the amplitude changing with frequency in the entire frequency range, and *l* is the length of the signal *x*(*t*)’. The instantaneous frequency of IMF, which is obtained from the Hilbert transform, is well-localized in the time-frequency domain and reveals important characteristics of the signal.

### 2.2. Probabilistic Committee Machine

PCM is a group decision method which combines the results from the individual classifier and generates superior performance to any of the individual classifier acting alone. As mentioned previously, RVM is selected for constructing the probabilistic fault classifier. To solve the multi-label classification problem effectively, RVM adopts a pairwise coupling strategy which is named PCRVM. Moreover, a new ensemble method is proposed to combine the output of each committee member. In the proposed ensemble method, the committee members should be assigned suitable weights since every member/classifier in the group usually has its own strength. The details of PCRVM algorithm and ensemble method are described in the following sections.

#### 2.2.1. Relevance Vector Machine

RVM is a statistical learning method utilizing Bayesian learning framework and popular kernels. In this research, predicting the posterior probability of each fault *t*_n_ for unseen symptoms **f** is conducted by RVM based on experimental data. Given a set of training data (**f**, **t**) = {**f**_n_,*t_n_*}, *n =* 1 to *N*, *t*_n_ ∈ {0, 1}, and *N* is the number of training data. It follows the statistical convention and generalizes the linear model by applying the logistic sigmoid function σ(y(f))=1/(1+exp(−y(f))) to the predicted decision y(**f**) and adopting the Bernoulli distribution for P(t|F), the likelihood of the data is written as:

(7) $$\begin{array}{r} P(t|F,\theta )=\prod_{n=1}^{N}\sigma \{y(f_{n};\theta {)}^{t_{n}}\}{\left[1-\sigma \{y(f_{n};\theta )\}\right]}^{1-t_{n}}\\ \mathrm{where}\;\;y(f;\theta )=\sum_{i=1}^{N}\theta_{i}K(f,f_{i})+\theta_{0} \end{array}$$

where $\theta ={\left(\theta_{0},\theta_{1},...,\theta_{N}\right)}^{T}$ is a weight vector and *K* is a kernel function. In the open literatures, three kinds of kernel functions, radial basis function (RBF), polynomial, and Gaussian kernels, are available. Among these kernel functions, Gaussian kernel is the most popular kernel function in RVM to deal with the issue of classification for industrial applications [34].

The optimal weight vector $\theta^{*}$ for the given dataset needs to be computed so as to maximize the probability *P*(θ|**t**, **F**, ***α***) ∝ *P*(**t**|**F**, θ)*P*($\theta^{*}$|***α***), with ***α*** **=** **[***α*_0_*, α*_1_*, …, α_N_***]** a vector of *N* + 1 hyperparameters. However, the weights cannot be determined analytically. Thus, the following approximation procedure is chosen, which is based on Laplace’s method:

(1) For the current fixed values of ***α***, the most probable weights $\theta_{\mathrm{MP}}$ are found. Since *P*(θ|**t**, **F**, ***α***) ∝ *P*(**t**|**F**, θ)*P*(θ|***α***), this step is equivalent to the following maximization.
  (8) $$\begin{array}{ll} \theta_{\mathrm{MP}} & =\arg\;\underset{\theta }{\max}\log\{P\left(t|F,\theta \right)P(\theta |\alpha )\}\\ & =\arg\;\underset{\theta }{\max}\{\sum_{n=1}^{N}[t_{n}\log d_{n}+(1-t_{n})(1-\log d_{n})]-\frac{1}{2}\theta^{T}A\theta \} \end{array}$$
  where $d_{n}=\sigma \{y(f_{n};\theta )\},A=\mathrm{diag}(\alpha_{0},\alpha_{1},...,\alpha_{N})$.
(2) Laplace’s method is simply a Gaussian approximation to the log-posterior around the mode of the weights $\theta_{\mathrm{MP}}$. Equation (8) is differentiated twice to give:
  (9) $$\nabla_{\theta }\nabla_{\theta }\log P(\theta |t,F,\alpha ){|}_{\theta_{MP}}=-(\Phi^{T}B\Phi +A)$$
  where $B=\mathrm{diag}(\beta_{1},\beta_{2},...,\beta_{N})$ is a diagonal matrix with $\beta_{n}=\sigma \{y(f_{n};\theta )\}[1-\sigma \{y(f_{n};\theta )\}]$ and Φ is a *N* × (*N* + 1) design matrix with $\Phi_{nm}=K(f_{n},f_{m-1})$ and $\Phi_{n0}=1$, *n* = 1 to *N*, and *m* = 1 to *N* + 1. By inverting Equation (9), the covariance matrix $\sum ={\left(\Phi^{T}B\Phi +A\right)}^{-1}$ can be obtained.
(3) The hyperparameter vector ***α*** is updated using an iterative re-estimation equation. Firstly, *α_i_* is randomly guessed, then $\gamma_{i}=1-a_{i}\sum_{ii}$ is calculated, where $\sum_{ii}$ is the *i*th diagonal element of the covariance matrix ∑⋅ Then, *α_i_* is re-estimated as follows:
  (10) $$\alpha^{new}=\frac{\gamma_{i}}{u_{i}^{2}}$$
  where $u=\theta_{\mathrm{MP}}=\sum \Phi^{T}Bt$. The first step is to set $\alpha_{i}\leftarrow \alpha_{i}^{new}$ and then $\gamma_{i}$ and $\alpha_{i}^{new}$ are re-estimated again until convergence. Finally, $\theta =\theta_{\mathrm{MP}}$ is set, so that the classification model $y(f;\theta )=\sum_{i=1}^{N}\theta_{i}K(f,f_{i})+\theta_{0}$ is obtained.

#### 2.2.2. Pairwise-Coupled Relevance Vector Machine as Committee Member

**Figure 2:**
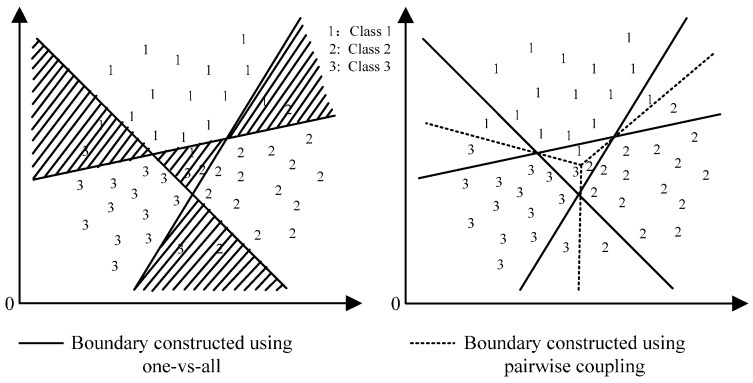
Indecisive regions (shaded regions) using one-vs-all (**left**) and pairwise coupling (**right**).

**Figure 3:**
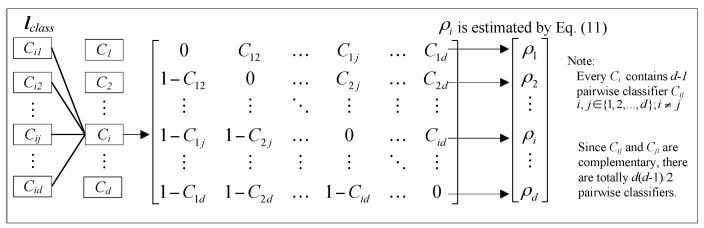
Pairwise coupling strategy of probabilistic classification.

The traditional machine learning methods are designed only for the issue of binary classification, in which the output is either positive (+1) or negative (−1). However, most practical problems are multi-classification as well as probabilistic output. Usually, one-*versus*-all is employed to deal with multi-classification problems. The one-*versus*-all strategy constructs a group of classifiers ***l***_class_ = [*C*_1_, *C*_2_, …, *C_d_*] in a *d*-label classification problem. The one-*versus*-all strategy is simple and easy to implement, however, it generally gives a poor result [29,35] since one-*versus*-all does not consider the pairwise correlations which causes a much larger indecisive region than the pairwise coupling strategy (using one-*versus*-one) as showed in Figure 2. The pairwise coupling strategy also constructs a group of classifiers ***l***_class_ = [*C*_1_, *C*_2_, …, *C_d_*] in a *d*-label classification problem. However, each ***C****_i_* = [*C_i_*_1_, *C_i_*_2_, …, *C_id_*] is composed of a set of *d* − 1 different pairwise classifiers *C_ij_*, i≠j. Since *C_ij_* and *C_ji_* are complementary, there are totally *d*(*d* − 1)/2 classifiers in ***l***_class_ as shown in Figure 3. To solve the multi-classification and probabilistic output problems, a pairwise coupling strategy is adopted for the RVM and PNN classifiers. The strategy combines all the outputs of every pair of classes to re-estimate the overall probability for a new instance.

There are several available methods for pairwise coupling strategy [29], which are, however unsuitable for simultaneous-fault diagnosis because of the constraint $\sum^{\text{​}}\rho_{i}=1$. Where $\rho_{i}$ is the probability of the *i*th label. Note that the nature of simultaneous-fault diagnosis is that $\sum^{\text{​}}\rho_{i}$ is unnecessarily equal to 1. Therefore, the following simple pairwise coupling strategy for simultaneous-fault diagnosis is proposed. Every $\rho_{i}$ is calculated as:

(11) $$\rho_{i}=C_{i}(x)=\frac{\sum_{i=1:i\neq j}^{d}n_{ij}C_{ij}(x)}{\sum_{j=1:i\neq j}^{d}n_{ij}}=\frac{\sum_{j=1:i\neq j}^{d}n_{ij}\rho_{ij}}{\sum_{j=1:i\neq j}^{d}n_{ij}}$$

where *n_ij_* is the number of training feature vectors with either the *i*th or *j*th label. Hence, the probability can be accurately estimated from $\rho_{ij}=C_{ij}\left(x\right)\;$because the pairwise correlation between the labels is taken into account. With the above pairwise coupling strategy, the proposed probabilistic committee member, PCRVM, could estimate the probability vector ρ in a high level of accuracy.

After designing the pairwise coupling strategy for each probabilistic classifier, a new ensemble method is proposed to combine the result from each committee member with optimal weight.

#### 2.2.3. Ensemble Method

One of the most frequently used ensemble methods is weighted averaging. In this method, every committee member has an appropriate weight related to its ability. However, the weighted averaging method cannot give a fair result when it deals with the issue of unbalanced committee member sensitivities to faults. For example, when the committee member 1 is not trained by a dataset with the fault *d*_5_, the fault *d*_5_ usually cannot be predicted by the committee member 1, which is demonstrated in Table 2. However, the weight averaging method still uses the unpredictable output to calculate the overall average, resulting in an unfair or unpredictable result.

To overcome the above problem, a novel ensemble method with optimal weights and predefined null outputs is proposed which is given by Equation (12). In Equation (12), $\rho_{j-i}$ is set to be zero when the *j*th classifier cannot make a diagnosis for the *i*th fault label (*i.e.*, the *j*th classifier is not trained by the *i*th single-fault). In this way, the proposed method can overcome the problem of the traditional weighted averaging method, which is one of main contributions of this research. The probability of the *i*th fault is expressed as:

(12) $$\begin{array}{l} P_{i}=\frac{\sum_{j=1}^{k}w_{j-opt}\rho_{j-i}}{\sum_{j=1}^{k}f(w_{j-opt})}\;,i=1,2,...,d\;\&\;j=1,2,...,k\\ \mathrm{subject}\;\mathrm{to}\;f(w_{j-opt})=\left\{ \begin{array}{l} w_{j-opt}\\ 0\;:\;if\;\rho_{j-i}=0 \end{array}\right. \end{array}$$

where *w_j-opt_* is the optimal weight for the *j*th committee member, *w_j-opt_* ∈[0, 1], *j* = 1 to *k*, where *k* is the number of committee members, and the sum of *w_j-opt_* is not equal to 1. $\rho_{j-i}\in \left[0,\;1\right]$ is probability estimated from the *j*th classifier for the *i*th single-fault, *i* = 1 to *d* where *d* is the total number of detectable single-faults. Finally, the probabilistic outputs of classifiers are combined with optimal weights to generate the probability vector **P** = [P_1_, P_2_, ...*,* P*_d_*].

**Table 2:** Issue of weighted averaging method for balanced and unbalanced committee member sensitivities to gearbox faults.

Balanced Member Sensitivities to Gearbox Faults	Committee Member 1	Committee Member 2	Average Output Probability (*P*_3_) for *d*_3_
Fault *d*_3_	trained	trained	$P_{3}=\frac{w_{1}\rho_{1-2}+w_{2}\rho_{2-2}}{w_{1}+w_{2}}\in [0\;1]$ *P*_3_ is a reasonable result
Output probability for *d*_3_ for an unseen case	$\rho_{1-3}\in [0\;1]$	$\rho_{2-3}\in [0\;1]$	
**Unbalanced Member Sensitivities to Gearbox Faults**	**Committee Member 1**	**Committee Member 2**	**Average Output Probability (*P*_5_) for *d*_5_**
Fault *d*_5_	Unable to train	trained	$P_{5}=\frac{w_{1}\rho_{1-5}+w_{2}\rho_{2-5}}{w_{1}+w_{2}}$ *P*_5_ is an unfair/unpredictable result
Output probability for *d*_5_ for an unseen case	$\rho_{1-5}$ is unpredictable	$\rho_{2-5}\in [0\;1]$	

Remark: w_1_ and w_2_ are weights for Committee members 1 and 2 respectively; P_3_ and P_5_ are average output probabilities for d_3_ and d_5_ respectively.

**Figure 4:**
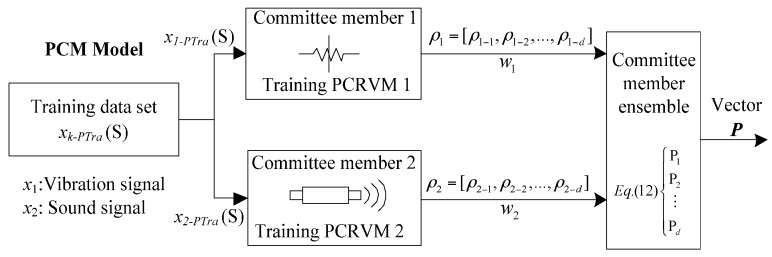
Procedure for training probabilistic committee machine.

In this application, the processed training datasets *x_k-PTra_*, are employed to train probabilistic classifiers (PCRVM) respectively. The workflow of the PCM is shown in Figure 4.

### 2.3. Parameter Optimization

The probability vector **P** = [P_1_, P_2_, …, P*_d_*] can be provided to the user as a quantitative measure for reference and further processing. However, human experts generally cannot identify the number of simultaneous-faults directly based on the output probability of each fault. Therefore, a decision threshold (DT) *ε* is introduced to identify the simultaneous-faults from **P** such that:

(13) $$y_{i}=\{\begin{array}{cc} 0 & \\ 1 & \mathrm{if}\;P_{i}\geq \varepsilon \end{array}$$

where ε∈[0 1] and 1 denotes that the corresponding fault occurs. For example, given an unseen input *x*, if **P** = [0.72, 0.42, 0.51, 0.81, 0.39] and *ε* = 0.5, then ***y*** = DT(**P**) = [1, 0, 1, 1, 0]. Therefore, the unseen *x* is diagnosed as a simultaneous-fault for the labels (1, 3, 4).

Obviously, the weight and the decision threshold are the major factors affecting the classification accuracy. By reviewing the literature [30,31], it is seen that PSO has the same effectiveness as a typical optimization method, genetic algorithms, in finding the global optimal solution, but with better computational efficiency. Hence, PSO is adopted to determine the best weights *w_opt_* and decision threshold *ε_opt_* in this study.

#### Particle Swarm Optimization

PSO is a population-based optimizer. The population is regarded as a swarm and the individuals are considered as particles. For an *z*-dimensional search space and a swarm consisting of *H* particles, the *i*th particle can be represented by an *z*-dimensional vector ***u****_i_* = (*u_i_*_1_, *u_i_*_2_, …, *u_i_*_z_), the velocity of this particle can be an *z*-dimensional vector ***v****_i_* = (*v_i_*_1_, *v_i_*_2_, …, *v_i_*_z_), and the best previous position encountered by this particle can be described as ***p****_i_* = (*p_i_*_1_, *p_i_*_2_, …, *p_i_*_z_). Let *g* represent the index of the particle that attains the best previous position among all the particles in the swarm. Then, the swarm is manipulated in accordance with the following equations:

(14) $$v_{i}(j+1)=W_{f}v_{i}(j)+q_{1}r_{1}[p_{i}(j)-u_{i}(j)]+q_{2}r_{2}[p_{g}(j)-u_{i}(j)]$$

(15) $$u_{i}(j+1)=u_{i}(j)+v_{i}(j+1)$$

where *i* is the particle index *i* = [1, 2, …, *H*], *W_f_* is the weight factor, *q*_1_ and *q*_2_ are positive constants, *r*_1_ and *r*_2_ are the random numbers selected between [0, 1]. The selection of the above parameters was presented in [36]. With reference to the literature, Table 3 shows the PSO parameters selected for this case study.

**Table 3:** PSO parameters.

Number of generations	1000
Population size	50
*W_f_*	0.9
*q*_1_	2
*q*_2_	2

**Figure 5:**
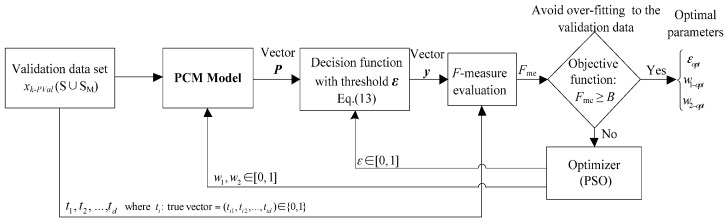
Procedure for optimization of committee member weights and decision threshold.

To evaluate the fitness of each iteration, a common evaluation method called *F*-measure [37] and an objective function described in Section 2.4 are employed. The procedure of the proposed PSO approach is illustrated in Figure 5, which is performed in three steps:

(1) Initializing the parameters of PSO: The candidate weight (*w*_1_, *w*_2_) and decision threshold are randomly selected from interval [0, 1].
(2) Calculating the output of *F*-measure: Following the procedure in Figure 5, the candidate weight and decision threshold are entered into the PCM model and Equation (13), respectively.
(3) Comparing the output of *F*-measure with the objective function: If the *F*-measure satisfies the objective function, the corresponding weights and decision threshold are taken as optimal parameters, otherwise PSO updates the weights and decision threshold based on Equations (14) and (15), and then repeats Steps 2 and 3. When it reaches the present number of generation or satisfies the objective function, the corresponding weights and decision threshold of the highest output of *F-*measure are taken as optimal parameters.

### 2.4. Performance Evaluation

**Figure 6:**
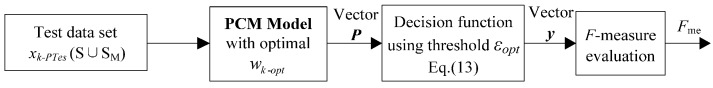
Evaluation of proposed framework.

The traditional performance evaluation of classifiers only considers exact matching of the decision vector **y** against the true vector **t**. This evaluation is however unsuitable for simultaneous-fault diagnosis where partial matching is preferred. *F*-measure is mostly used as a performance evaluation for information retrieval systems where a document may belong to a single or multiple tags simultaneously, which is very similar to the current study. By using *F*-measure, the evaluation of both single-fault and simultaneous-fault test cases can be fairly examined. The definition of *F*-measure is given in Equation (16). The larger the *F*-measure value, the higher the diagnostic accuracy is:

(16) $$F_{me}=\frac{2\sum_{j=1}^{d}\sum_{i=1}^{N_{t}}y_{ij}t_{ij}}{\sum_{j=1}^{d}\sum_{i=1}^{N_{t}}y_{ij}+\sum_{j=1}^{d}\sum_{i=1}^{N_{t}}t_{ij}}\in [0,\;1]$$

where ***y****_i_* = [*y_i_*_1_, *y_i_*_2_, …, *y_id_*] and **t***_i_* = [*t_i_*_1_, *t_i_*_2_, …, *t_id_*] are the predicted decision vector and the true decision vector respectively, for *j =* 1 to *d* and *i =* 1 to *N_t_* and ∀*y_ij_*, *t_ij_* ∈ [0, 1]. *N_t_* is the number of single-fault and simultaneous-fault test patterns. For optimization of the weights and decision threshold, *F_me_* also serves as an important parameter in an objective function. In order to avoid over-fitting to the validation dataset and achieve high diagnostic accuracy, the objective function is specifically defined as:

(17) $$F_{me}\geq B$$

where *B* is the preset optimal accuracy of *F*-measure and *B* lies between 0 and 1. In this study, *B* is set to be 0.9 as a trial. Figure 6 summarizes the evaluation process for the proposed diagnostic framework.

## 3. Experimental Setup and Data Preprocessing

To verify the effectiveness of the proposed framework, experiments were carried out. The detail of the experimental set up is presented in the following subsections. All the proposed methods were implemented by using MatLab R2008a and executed on a computer with a Core 2 Duo E6750 @ 2.13 GHz with 4 GB RAM.

### 3.1. Test Rig and Sample Data Acquisition

**Figure 7:**
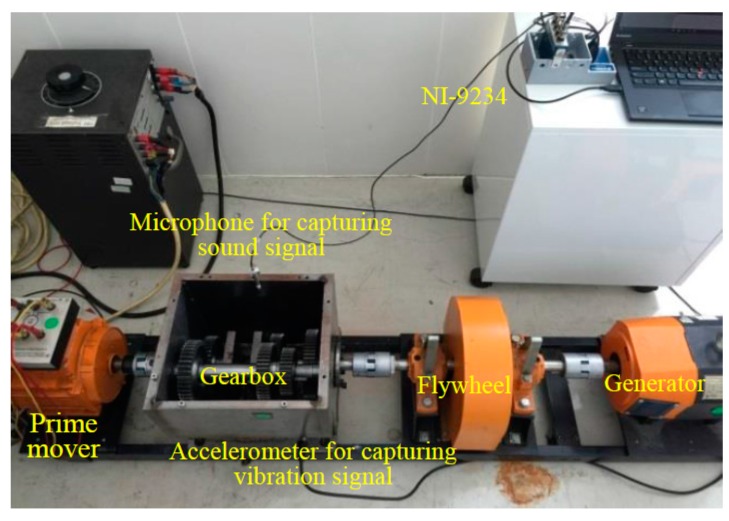
Collection of fault patterns from a rotating machinery.

The experiments were performed on a test rig as shown in Figure 7, which can simulate most of the faults in a gearbox. In this study, some common gearbox faults, including gear faults, bearing faults, and structural faults, are introduced. In the experiments, the gear faults include a broken tooth with whole tooth damage, a chipped tooth with 1/4 tooth damage, and a gear crack with a 5 mm crack on the tooth face, whereas the bearing faults include medium wear on the rolling elements and outer races. The structural faults contain unbalance, looseness, and misalignment, which are simulated by respectively adding one eccentric mass on the output shaft, unfastening some screws of the gearbox, and adjusting one height of the gearbox with shims. In the test rig, the signal acquisition module (NI 9234) with accelerometers and a microphone acquires the vibration and sound signals, respectively. The accelerometer is used to record the vibration signals along the vertical direction. In this study, a total of 12 cases, including eight single-faults and four simultaneous faults which are described in Table 4, are simulated in the test rig in order to generate sample training and test datasets. According to practical experience, a machine cannot be operated if there are too many faults at the same time. Therefore, the type of simultaneous faults is an experimental selection in this case study. Besides, the relationship between simulated faults and signal types is presented in Table 5, which explains that one kind of signal can only detect a limited number of faults. For example, previous experiments have found that the vertical vibration signal cannot be used to detect *d*_4_ and *d*_5_ because the loading on the tapered roller bearing along the vertical direction is insignificant. Moreover, the sound signal is relatively unaffected by structural resonance [38], so the structural failures (*d*_1_, *d*_2_ and *d*_3_) cannot be easily detected using the sound signal. To extend the number of detectable faults and enhance the reliability of the fault diagnostic system, the vibration and sound signals are therefore simultaneously employed to diagnose the simultaneous-faults in the gearbox.

**Table 4:** Description of single-faults and simultaneous-faults.

Case No.	Single-Faults	Case No.	Simultaneous-Faults
*d*_1_	Unbalance	*si*_9_	Broken gear tooth & Chipped tooth
*d*_2_	Looseness		
*d*_3_	Mechanical misalignment	*si*_10_	Chipped tooth & Bearing with worn outer race
*d*_4_	Bearing with worn rolling elements		
*d*_5_	Bearing with worn outer race	*si*_11_	Broken gear tooth & Bearing with worn rolling elements
*d*_6_	Broken gear tooth		
*d*_7_	Gear crack	*si*_12_	Bearing with worn rolling elements & Bearing with worn outer race
*d*_8_	Chipped tooth		

**Table 5:** Relationship of single-faults and signal types.

	*d*_1_	*d*_2_	*d*_3_	*d*_4_	*d*_5_	*d*_6_	*d*_7_	*d*_8_
Vertical vibration	√	√	√			√	√	√
Sound				√	√	√	√	√

To construct and test the proposed diagnostic framework, the samples for each single fault and simultaneous fault were repeated 200 times under two testing conditions (800 rpm and 1500 rpm). Each time, 1 s of raw signal, including the vibration and sound signals,wa simultaneously recorded with a sampling rate of 25.6 kHz. In other words, one case of each type of signal has 25,600 sampling data points. For each type of signal *x_k_* (*k* = 1, 2), there are 1600 single-fault sample data (*i.e.*, eight kinds of single faults × 200 samples) and 800 simultaneous fault sample data (*i.e.*, four kinds of simultaneous faults × 200 samples). In order to evaluate the diagnostic performance for both single faults and simultaneous faults, each sample data is divided into different subsets as shown in Table 6.

**Table 6:** Division of sample dataset into different subsets.

	Type of Dataset	Single-Faults (1600)	Simultaneous-Faults (800)
Raw sample data (*x_k_*)	Validation dataset	*D_k-Val_* (800)	*D_k-Val_* (600)
	Training dataset	*D_k-Tra_* (600)	
	Test dataset	*D_k-Tes_* (200)	*D_k-Tes_* (200)
After feature extraction	Validation dataset	*D_k-PVal_* (800)	*D_k-PVal_* (600)
	Training dataset	*D_k-PTra_* (600)	
	Test dataset	*D_k-PTes_* (200)	*D_k-PTes_* (200)

### 3.2. Data Processing and Signal De-Noising in Case Study

In order to obtain the feature vector, the IMF energy pattern based on HHT is calculated with the following steps: (1) signal de-noising; (2) IMF component selection; and (3) IMF energy pattern calculation.

(1) *Signal de-noising*. In the signal de-noising phase, the mother wavelet and the level of decomposition *L* are selected according to a trial-and-error method. In this case study, four Daubechies wavelets (Db3, Db4, Db5, and Db6) are tried and the range of *L* is set from 3 to 5. Moreover, the soft threshold *T* is equal to 4.476 according to the equation $T=\sqrt{2\;\text{log}\;\left(\text{length}\;x\left(t\right)\right)}$. The effectiveness of de-noising using Db wavelets is verified by using signal to noise ratio (SNR) which is given as follows:

(18) $$\mathrm{SNR}=10\times \log_{10}(\frac{S_{\sigma }}{N_{\sigma }})$$

where $S_{\sigma }$ and $N_{\sigma }$ are the standard deviation of de-noised signal and noise signal respectively. A large value of SNR means more noise is eliminated. Considering the sound signal of *d*_6_ as an example, the de-noised result is shown in Table 7. It demonstrates that the SNR of Db5 with Level 3 is the highest, so it is suitable to de-noise the signal.

**Table 7:** Signal to noise ratio under different combinations of Db wavelets.

SNR	Level 3	Level 4	Level 5
Db3	12.689 db	11.041 db	10.191 db
Db4	12.690 db	11.090 db	10.207 db
Db5	12.847 db	11.126 db	10.271 db
Db6	12.720 db	11.118 db	10.272 db

(2) IMF *component selection*. After de-noising the signals, the IMFs of all de-noised signals are calculated by using EEMD in which the ensemble number and white noise amplitude of EEMD are set as 100 and 0.3 time of the standard deviation of the investigated signal respectively [33]. In this case study, EEMD decomposes the de-noised sound signal into ten IMFs and a residual signal. To select the proper number of IMFs, the correlation coefficient method [13] is used. The correlation coefficient between an IMF component *I_i_*(*t*) and its de-noised signal *x*(*t*)’ can be defined as:

(19) $$Coe_{x(t)\prime ,I_{i}(t)}=\frac{\sum_{i=1}^{M}\left(x(t)\prime -\bar{x})(I_{i}(t)-\bar{I_{i}}\right)}{\sqrt{\sum_{i=1}^{M}{\left(x(t)\prime -\bar{x}\right)}^{2}}\sqrt{\sum_{i=1}^{M}{\left(I_{i}(t)-\bar{I_{i}}\right)}^{2}}}$$

where $\bar{x}$ and $\bar{I_{i}}$ is the mean values of the *x*(*t*)’ and *I_i_*(*t*) respectively and *M* is the number of IMFs. A large $Coe_{x\left(t\right),I_{i}\left(t\right)}$ value means a high correlation between *I_i_*(*t*) and *x*(*t*)’, and also implies that *I_i_*(*t*) contains more fault information. A signal of correlation coefficients of de-noised sound signal of *d*_6_ is presented in Table 8 as a demonstration in which the correlation coefficient of IMF *I*_10_ is obviously smaller than the others. Thus, only the IMFs from levels 1–9 are considered to extract the energy pattern in this case study.

**Table 8:** Correlation coefficients of each IMF component for an example of de-noised signal of *d_6_*.

De-noised sound of *d*_6_		**IMF Component**									
		***I*_1_**	***I*_2_**	***I*_3_**	***I*_4_**	***I*_5_**	***I*_6_**	***I*_7_**	***I*_8_**	***I*_9_**	***I*_10_**
	Correlation coefficient	0.2054	0.2089	0.2132	0.2375	0.2489	0.3475	0.3134	0.2876	0.2273	0.0274

**Figure 8:**
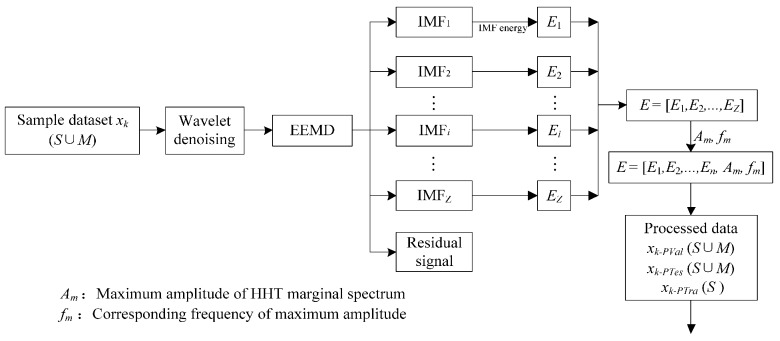
Flowchart of proposed feature extraction approach.

(3) IMF *energy pattern calculation*. In this case study, the energy patterns of selected IMFs are considered to extract the fault features. The energy of the *i*th IMF, *E_i_*, can be calculated by using the following equation:

(20) $$E_{i}=\sum_{j=1}^{n}\left[(j\cdot \Delta t)\cdot {\left|I_{i}(j\cdot \Delta t)\right|}^{2}\right]$$

where Δt is the time interval, *n* and *j* are the total number and index of data points respectively, and $I_{i}\left(j\cdot \Delta t\right)$ denotes the decomposition coefficient of the *i*th IMF at the moment of j·Δt. A nine-dimensional energy feature vector is extracted as *E =* [*E*_1_, *E*_2_, …, *E*_9_]. Furthermore, under different fault conditions, the HHT marginal spectra show various maximum values and corresponding frequencies in the patterns. To enrich the fault information, the maximum amplitude of a marginal spectrum of HHT, *A_m_*, and its corresponding frequency, *f_m_*, are added to the feature vector *E*. Therefore, the extracted feature vector is extended to an eleven-dimensional vector, which can be rewritten as *E =* [*E*_1_, *E*_2_, …, *E*_9_, *A_m_*, *f_m_*]. The procedure of data processing is illustrated in Figure 8.

## 4. Experimental Results and Discussion

### 4.1. Performance of Various Combinations of Feature Extraction Techniques

In the experiments, two typical feature extraction methods, fast Fourier transform (FFT) and wavelet package transform with principal component analysis (WPT + PCA) are compared with HHT. For those feature extraction methods, some settings are necessary. For the wavelet package transform (WPT), the Daubechies wavelet is the most popular one, so it is employed. In this case study, Db4 with level 4 decomposition is employed after carrying out many trials. Besides, two classification techniques are used to compare with the proposed PCM framework, including PCPNN and PCRVM. There are two hyper-parameters, spread S^*^ and width W* in the kernel function, which are necessary to be defined in PCPNN and PCRVM respectively. Meanwhile, PCRVM is employed as a committee member of PCM, so PCM and PCRVM share the same hyper-parameter width W*. By using a trial-and-error method, S^*^ and W* are set to be 0.3 and 0.64 respectively.

**Figure 9:**
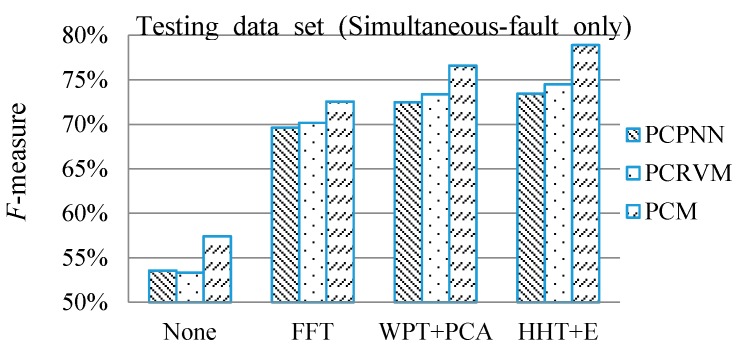
Diagnostic accuracies of different combinations of feature extraction techniques.

After determining the configurations of the feature extraction and classification techniques, the reasonable combinations of feature extraction techniques are tested as shown in Figure 9, in which the weight of each committee member and decision threshold are predefined as 1 (*i.e.*, *w*_1_ = *w*_2_ = 1) and 0.5 respectively. Note that PCPNN and PCRVM determine their *F*-measures by combining all the features extracted from vibration and sound signals as their input vectors, whereas PCM employs two PCRVM committee members to analyze the respective extracted features.

Figure 9 illustrates that the feature extraction techniques are effective. Taking the proposed PCM framework as an example, the feature extraction techniques, FFT, WPT + PCA, and Hilbert-Huang transform + energy pattern (HHT + E) give 14.12%, 18.18%, and 21.48% improvement respectively as compared with the method without any feature extraction. By using PCPNN and PCRVM as classifiers, the feature extraction methods also improve the diagnostic accuracy from 16.06% to 21.16% as compared with the method without feature extraction. Note that the classifiers only employ a training set of single-fault patterns to construct the classifiers while the performance is evaluated using simultaneous-fault test patterns. Figure 9 also indicates that no matter which classification technique is, HHT + E gives the best performance. The reason is that extracting the energy from HHT can reflect not only the energy amount of each IMF, but also the energy distribution of each IMF changing with time, which can provide more faulted component information. This result also verifies that the proposed feature extraction technique (HHT + E) is effective to extract the features of single-faults from simultaneous-fault patterns of the gearbox.

### 4.2. Result and Discussion of Optimization Approach

After selecting HHT + E as feature extraction technique, the extracted features are employed to construct and train the committee machine. Then, PSO and Equations (16) and (17) are employed to determine the best *w_opt_* for each committee member and decision threshold *ε_opt_*. The optimized weights and threshold as well as their corresponding *F_me_* are shown in Table 9 in which the optimal weight for the first committee member *w*_1_ (0.7752) is higher than that of *w*_2_. In other words, the committee member trained by vertical vibration signal shows a great impact on the simultaneous-fault diagnosis. The main reason is that the sound signal is easily interfered by background noise. It implies that the first committee member is assigned with greater weight by PSO in order to make the output satisfying the objective function. Table 9 also illustrates that the proposed optimization framework can improve the diagnostic accuracy by 3.82% as compared with the empirical decision threshold of 0.5 and identical weights (*w*_1_ = *w*_2_ = 1) under the same feature extraction technique and simultaneous-fault test dataset. It means that the proposed optimization framework is effective.

**Table 9:** Selection of optimal weights and decision threshold using PSO.

Classifier	No. of Features	Optimization Method	Decision Threshold	Weights	*F_me_* Based on Simultaneous-Fault Test Dataset
PCM	Vibration = 11 Sound = 11	-	0.5	*w*_1_ = 1 *w*_2_ = 1	0.7890
PCM	Vibration = 11 Sound = 11	PSO	0.7583	*w*_1_ = 0.7752 *w*_2_ = 0.6991	0.8272

Remark: Feature extraction method is based HHT + E.

### 4.3. Overall Evaluation of Proposed Framework

To verify the effectiveness of the proposed PCM diagnostic framework, the aforesaid two single probabilistic classifiers are compared with the proposed framework based on the optimal weights and decision threshold obtained by PSO. The experimental result of *F*-measure is shown in Table 10. Compared with PCPNN and PCRVM, the training time and average fault detection time of PCM are the longest, 36.189 s and 17.8574 s, respectively, while the result shows the diagnostic accuracy of PCM outperforms PCPNN and PCRVM by 5.24% and 4.18% respectively under the same test dataset of simultaneous-faults. Note that the training time of PCM is only based on the training dataset of single fault patterns; the average fault detection time of PCM relies on calculating the average time of test datasets of single, simultaneous and overall faults. Table 10 also reveals the proposed framework achieves the best accuracy for single faults (94.60%) and overall faults (89.24%) which include both single and simultaneous fault patterns. The main reason is that the committee members in the proposed framework are trained with different types of signals. In this way, each committee member becomes different from each other, which can improve the classification accuracy of the ensemble. For example, considering an ensemble of *k* trained classifiers [*C*_1_, *C*_2_, ..., *C_k_*], if the classifiers are trained using different subsets and their errors are uncorrelated, then even when *C_i_* is wrong, most of the other classifiers *C_j_* (where *i* ≠ *j*) may still be correct.

In a nutshell, the proposed framework is an effective approach to detect the simultaneous-faults without costly simultaneous-fault training patterns. Moreover, the proposed method employs vibration and sound signals to train the diverse committee members, which can ensure the diagnostic result to be more reliable and accurate. Therefore, it can be concluded that the proposed framework is an effective technique to overcome both challenges in fault diagnosis of the gearbox.

**Table 10:** Evaluation result of PCM, PCPNN and PCRVM.

Classifier	Feature Number	Decision Threshold	Optimal Weight	Accuracies for Test Cases (*F_me_*)			
				Single- Faults	Simultaneo- Us-Faults	Overall- Faults	Average Fault Detection Time (s)
PCPNN	11 + 11 = 22	0.6830	-	0.9163	0.7717	0.8563	8.8014
PCRVM	11 + 11 = 22	0.6754	-	0.9141	0.7823	0.8642	9.7685
PCM	Vibration = 11 Sound = 11	0.7583	*w*_1_ = 0.7752 *w*_2_ = 0.6991	0.9460	0.8241	0.8924	17.8574

Remark: Feature extraction method is based on HHT + E.

## 5. Conclusions

In this paper, a new framework, which combines signal de-noising, feature extraction, probabilistic committee machine, parameter optimization and *F-*measure, has successfully been developed to overcome the challenges of simultaneous fault diagnosis and multiple signal analysis in a gearbox. In consideration of the features of vibration and sound signals in this application, DWT and HHT + E are used for signal de-noising and feature extraction, respectively, so that the diagnostic system can effectively capture the single fault components from the noise-polluted simultaneous fault patterns. It implies that the acquisition of large amount of simultaneous fault signals can be avoided. Moreover, PSO is effective for optimizing the weight of each committee member and decision threshold in the PCM framework. To verify the effectiveness of the proposed probabilistic committee machine and make a comparison, the single probabilistic classifiers, PCPNN and PCRVM, are also employed to diagnose the simultaneous faults. Although the results show that those machine learning methods can diagnose the simultaneous faults in the gearbox, it is found that the proposed PCM framework is superior to the single classifiers. Therefore, the proposed PCM framework is suitable to detect the simultaneous faults in the gearbox.

In practice, most mechanical faults can be diagnosed by analyzing vibrations, sounds, currents, oil debris and temperature signals. As the number and type of committee members in the proposed framework can be adjusted by the user, the proposed framework can be applied to other similar diagnostic applications.
